# Angiotensin II directly regulates intestinal epithelial NHE3 in Caco2BBE cells

**DOI:** 10.1186/1472-6793-9-5

**Published:** 2009-04-01

**Authors:** Mark W Musch, Yan Chun Li, Eugene B Chang

**Affiliations:** 1 Department of Medicine, University of Chicago, Chicago, IL 60637, USA

## Abstract

**Background:**

Angiotensin II (AII) effects on intestinal Na^+ ^transport may be multifactorial. To determine if AII might have a direct effect on intestinal epithelial Na^+ ^transport, we investigated its actions on Na^+ ^transport in human intestinal epithelial Caco2BBE cells.

**Results:**

AII increased apical (brush border) sodium-hydrogen exchanger (NHE)-3, but not NHE2, activity within one hour. Similarly, only apical membrane NHE3 abundance increased at 1–2 hours without any change in total NHE3 protein abundance. From 4–48 hours, AII stimulated progressively larger increases in apical NHE3 activity and surface abundance, which was associated with increases in NHE3 protein expression. At 4–24 hours, NHE3 mRNA increases over baseline expression, suggesting increased gene transcription. This was supported by AII induced increases in rat NHE3 gene promoter-reporter activity. AII induction of NHE3 was blocked by the AII type I receptor antagonist losartan. Acute changes in AII-induced increases in NHE3 exocytosis were blocked by a phospholipase C inhibitor, an arachidonic acid cytochrome P450 epoxygenase inhibitor, as well as phosphatidylinositol 3 kinase (PI3K) inhibitors and Akt inhibitor, partially blocked by a metalloproteinase inhibitor and an EGF (epidermal growth factor) receptor kinase inhibitor, but not affected by an inhibitor of MEK-1 (MAPKK-1, mitogen activated protein kinase kinase-1).

**Conclusion:**

We conclude that angiotensin II has a direct role in regulating intestinal fluid and electrolyte absorption which may contribute to its overall effects in regulation systemic volume and blood pressure. AII activates several key signaling pathways that induce acute and chronic changes in NHE3 membrane trafficking and gene transcription.

## Background

The octapeptide angiotensin II (AII) has diverse effects and regulates organismal blood pressure through many mechanisms, including effects on renal and intestinal fluid and electrolyte transport and changes in vascular smooth muscle tone. Through these mechanisms, AII increases plasma volume and vasoconstriction, which contribute to its effect on blood pressure. In the kidney, in addition to stimulation of Na^+ ^reabsorption through increasing aldosterone release, AII also increases Na^+ ^transport at the proximal convoluted tubule through direct stimulation of apical sodium/hydrogen exchanger (NHE) activity [1-4], in part mediated by direct action on proximal tubular AII receptors [5-8]. In the GI tract, AII increases activity and expression of colonic electrogenic Na^+ ^channels [9,10], small intestinal electroneutral Na^+ ^absorption [11-13], modulates colonic K^+ ^transport [14], and may also induce HCO3^- ^secretion [15-17]. However the precise mechanism(s) underlying these effects remain incompletely understood. For some studies, the effects of AII on transport have been introduced vascularly [11,12] and therefore the effects could be direct or indirect, such as AII-induced alterations of enteric nervous control of ion transport or alterations of regional blood flow. Aldosterone is also thought to be involved in AII-induced sodium absorption in the GI tract, which targets the epithelial sodium channel [12]. However, AII binding sites have been demonstrated in membranes from intestinal epithelial cells [18] and AII affects growth and proliferation of cultured small intestinal epithelial cells [19-21], suggesting direct intestinal effect of AII.

The present studies demonstrate that AII increases, in an aldosterone independent fashion, activity and expression of the apical sodium/hydrogen exchanger NHE3, but not NHE2, in cultured Caco2BBE cells. Because apical membrane NHEs of the intestine are the major mediators of non-nutrient dependent absorption of Na^+ ^[22,23], these effects can potentially contribute to overall maintenance of metabolic balance and blood presssure. These effects are mediated by type I AII receptors through pathways that are dependent on phospholipase C, epoxygenase metabolism of arachidonic acid, phosphatidyl inositol 3 kinase and Akt, and partially on metalloproteinase activity and stimulation of the EGF receptor. These studies therefore provide compelling evidence of direct regulation of apical NHE3 in intestinal epithelial cells by AII.

## Results

### Angiotensin II increases NHE3, but not NHE2, activity and membrane insertion acutely and in long term

**Figure 1:**
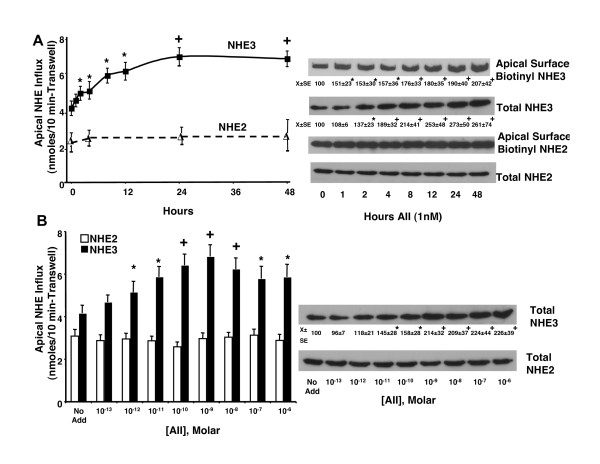
**Angiotensin II increases NHE3 but not NHE2 in Caco2BBE cells**. (A) Monolayers were treated basolaterally with (A) 1 nM AII for varying times or (B) varying concentrations of AII for 48 hours. Apical NHE2 and NHE3 activities were measured as described in Materials and Methods. Apical surface NHE3 and NHE2 were measured using apical surface biotinylation. Total NHE expression was measured in whole lysates. Fluxes and images are representative of four separate experiments. Flux values are means ± SEM. Densitometric values were calculated setting intensity for the zero time value (A) or no addition (B) to 100% in each experiment and calculating densitometric changes (decreases or increases) of other groups relative to these values. * P < 0.05 + P < 0.01 compared with untreated zero time control by analysis of variance using a Bonferroni correction.

Caco2BBE cell monolayers were treated on the basolateral side with 1 nM angiotensin II for times ranging from 1–48 hours. Apical NHE activities were measured as ^22^Na^+ ^uptake sensitive to amiloride analogs HOE694 (30 μM to inhibit NHE2) or DMA (500 μM to inhibit both NHE3 and NHE2), as previously described [24]. NHE2 and NHE3 activities were defined as the HOE694-sensitive and -insensitive components of DMA-inhibited ^22^Na uptake, respectively. After two hours, 1 nM angiotensin II significantly increased apical NHE3, but not NHE2 activity (Figure 1A, left panel). The increased NHE3 activity was paralleled by increased apical surface abundance of NHE3, as assessed by apical surface biotinylation (Figure 1A right panel). In previous studies [25] we had demonstrated that the conditions for apical surface biotinylation do not result in biotinylation of either basolateral surface proteins or intracellular proteins. Equivalent protein amounts (500 μg) were used for apical surface biotinylation and total NHE analyses (50 μg). Apical addition of 1 nM AII did not stimulate apical ^22^Na^+ ^transport at any time up to 48 hours (data not shown). Further increases in apical NHE3 activity were observed between 4–48 hours after AII stimulation, occurring in two 'phases'. From 1–4 hours, a smaller increase in apical NHE3 activity was observed with a progressive increase from 4 to 24 hours that was maintained for at least 24 hours. These changes were associated with increased apical surface NHE3 abundance. Within one hour, however, little increase in total NHE3 protein expression was observed and from 2–48 hours, NHE3 protein expression increased (Figure 1A). No changes were observed for apical surface or total NHE2 over this time (Figure 1A). AII increased NHE3 expression and activity at 24 hours in a concentration-dependent fashion with effects beginning at low pM concentrations and maximal effects near 1 nM, concentrations that are in the physiologic range (Figure 1B).

**Figure 2:**
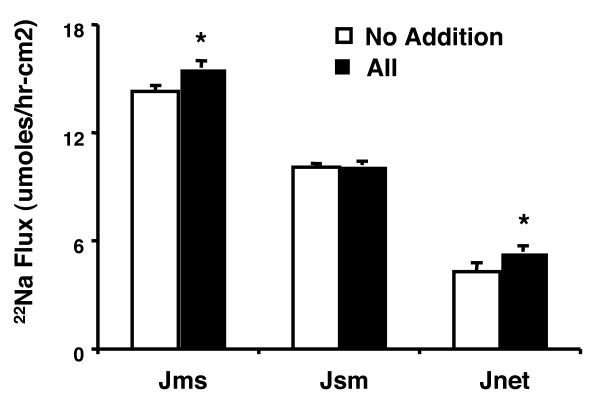
**Angiotensin II stimulates absorptive Na^+ ^flux in mouse jejunum**. Sections of mouse jejunum were mounted in Ussing chambers and matched for transepithelial electrical resistance (differed by less than 20%). One set was treated with 1 nM AII on the serosal side and mucosal (m) to serosal (s) and s-to-m fluxes measured over the next 30 min. Values are means ± SEM for six separate experiments. * p < 0.05 compared with untreated tissues by paired Student's T test.

To determine if AII stimulates Na^+ ^transport in native intestine, segments of mouse jejunum were mounted in Ussing chambers and transmural ^22^Na^+ ^fluxes measured. Addition of AII (added 15 min serosally before initiation of flux period) significantly increased the mucosal to serosal absorptive flux without change in the seriosal to mucosal flux, demonstrating that the AII-induced apical NHE3 activity observed in cultured Caco2BBE cells is also observed in native intestine (Figure 2). The increase in m-to-s flux is small, however, it should be noted that the incubations time with AII was limited due to the limited viability of mouse jejunum in Ussing chambers. AII was therefore added approximately 10 min after mounting tissues in the chambers and allowed to incubate with the mucosal strip for 15 min before initiating the 30 min flux period. Had the experimental conditions allowed longer incubations, we suspect that the AII effect would have been greater.

### AII stimulates transcription of the NHE3 gene

**Figure 3:**
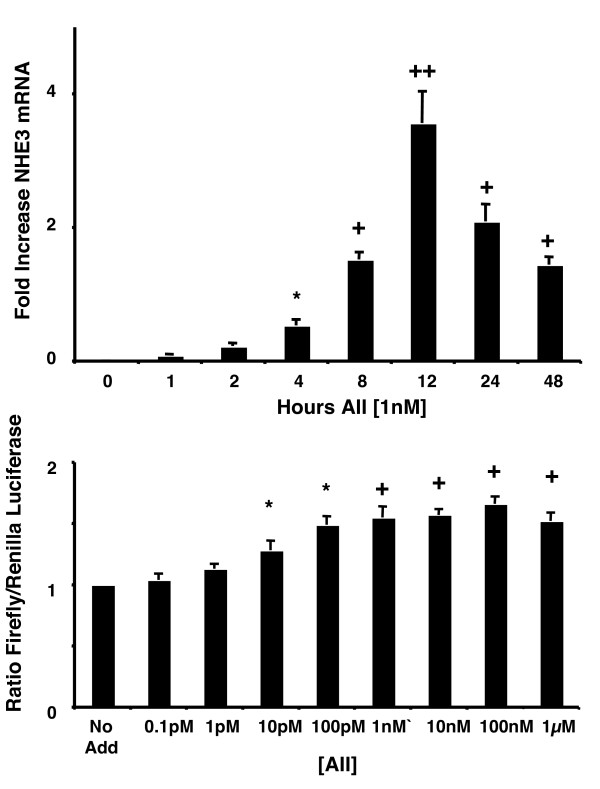
**Angiotensin II increases NHE3 gene transcription**. (A) Monolayers were treated with AII (1 nM, basolaterally) for varying times and RNA harvested and analyzed for NHE3 mRNA by real-time PCR. GAPDH was used as a constitutive mRNA control and NHE3 mRNA increases calculated by the ΔΔCt method (33). Values are means ± SEM for four separate experiments. * P < 0.05 + P < 0.01 ++ P < 0.001 compared with zero time untreated control by analysis of variance using a Bonferroni correction. (B) Monolayers were transfected with plasmids containing a 2200 bp segment of the rat NHE3 gene promoter linked to firefly luciferase cDNA and another with the thymidine kinase promoter linked to Renilla luciferase as a constitutive control. Cells were treated with AII (0–1 μM) 24 hours after transfection and monolayers were harvested and luciferase activities measured after 24 hours. Values are means ± SEM for four separate experiments. * p < 0.05 compared with untreated zero time control by analysis of variance using a Bonferroni correction.

To determine whether AII increased NHE3 transcriptionally, mRNA levels for NHE3 were measured by real time PCR. AII increased NHE3 mRNA as early as 2 hours after treatment and this effect was maximal at 12 hours (Figure 3). To determine that the mRNA increase was indeed due to increased transcription and not message stabilization, luciferase reporter assays with a 2200 bp segment of the rat NHE3 gene promoter linked to firefly luciferase was used [26]. AII increased luciferase activity in a concentration dependent manner (Figure 3B), demonstrating that AII promoted NHE3 gene transcription.

### AII stimulation of NHE3 employs the type I receptor

**Figure 4:**
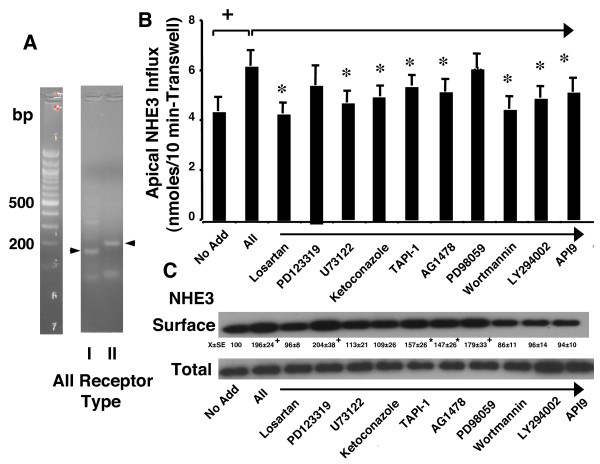
**Type I AII receptors mediate AII effects on NHE3 through activation of phospholipase C, epoxygenation of arachidonic acid, stimulation of the EGF receptor, and activation of phosphatidyl inositol 3 kinase and Akt**. (A) RNA was harvested from a cell monolayer, reverse transcribed, and analyzed for AII type I and II receptors by PCR. (B) To determine the mechanism of action of AII, cell monolayers were treated basolaterally with the type I receptor antagonist losartan (100 μM) or the type II receptor antagonist PD123319 (100 μM) or with inhibitors of phosholipase C (U73122, 30 μM), cytochrome P450 fatty acid epoxygenase (ketoconazole, 30 μM), a metalloproteinase inhibitor (TAPI-1, 100 μM), EGF receptor tyrosine kinase (Tyrphostin AG1478, 30 μM), MEK-1 (PD98059, 30 μM), phosphatidyl inositol 3 kinase (wortmannin, 30 μM or LY294002, 30 μM), Akt (API-1, 30 μM) all for 30 min prior to addition of AII (1 nM). Fluxes were measured 1 hr after basolateral addition of AII. Values are means ± SEM for four separate experiments. * P < 0.05 + P < 0.01 as designed for comparisons by analysis of variance using a Bonferroni correction. (C) Cells were treated as for flux studies in B, however, cells were used for apical surface biotinylation as described in Methods. Image shown is representative of those of four separate experiments. Densitometric values were calculated by setting the intensity for no addition to 100% in each experiment and calculating densitometric changes of other groups (decreases or increases) relative to this value. Values are means ± SE. * P < 0.05 + P < 0.01 as designed for comparisons by analysis of variance using a Bonferroni correction.

To determine which type AII receptors were expressed by Caco2BBE cells, mRNA was isolated and primers specific to the type I and II receptors were used for RT-PCR analyses. Both types of receptors were expressed by these cells (Figure 4A). To confirm that the PCR products were type I and II human AII receptors, PCR bands were subcloned and sequenced. Sequence of these PCR products was identical to the gene sequences, confirming expression of both receptor types. Therefore, to determine whether the acute stimulation of NHE3 by AII used the type I or II receptor, the receptor blockers losartan(type I) and PD123319 (type II) were used. Inhibition of type I but not type II receptors inhibited the AII stimulated apical Na^+ ^influx (Figure 4B) as well as AII stimulated exocytosis of NHE3 (Figure 4C).

To determine the mechanism of action, a panel of inhibitors were used for pathways known to be activated by AII or other G protein coupled receptors [20,21,27]. The following panel of inhibitors were tested: U73122, a phospholipase C inhibitor; ketoconazole, a cytochrome P450 inhibitor which blocks fatty acid epoxygenation; TAPI-1, a metalloproteinase inhibitor; tyrphostin AG-1478, an EGF receptor tyrosine kinase inhibitor; PD98059, a MEK-1 inhibitor; wortmannin and LY294002, phosphatidyl inositol 3 kinase inhibitors, and API-9, an Akt inhibitor. All of these agents except PD98059 inhibited the AII stimulation of NHE3 activity after 1 hour (Figure 4B), effects that were paralleled by their effects on AII stimulated apical surface NHE3 (Figure 4C).

To determine if the long term changes in NHE3 expression were also mediated by type I receptor stimulation, cells were pretreated with losartan or PD123319 and stimulated with AII for 24 hours and Na^+ ^fluxes measured. Inhibition of the type I receptor blocked the AII-stimulated Na+ flux increase while PD123319 had no effect (fluxes for each condition in nmoles/10 min-Transwell, n = 4, means ± SEM: no addition 4.08 ± 0.33, AII (1 nM) 6.59 ± 0.68, losartan + AII 4.29 ± 0.54, and PD123319 + AII 6.36 ± 0.79). Therefore the long term effects of AII on Caco2BBE NHE3 are also mediated by type I receptor stimulation.

## Discussion

The role of AII in regulation of blood pressure is well established, however, its actions are likely to occur through multiple mechanisms including effects on vascular smooth muscle and endothelium [28,29]. AII can also affect salt and water homeostasis through its actions on renal Na reabsorption [1,30]. In addition, AII stimulates aldosterone production by the adrenal gland that is a major regulator of renal and intestinal Na^+ ^transport [31]. The present studies demonstrate that AII has direct effects on intestinal epithelial Na^+ ^transport that are consistent with its desired effect to increase fluid absorption. Although AII has been noted to increase small intestinal Na^+ ^absorption previously [11,13], the present studies provide a more definitive analysis, demonstrating that this is due to stimulated exocytosis of the apical transporter NHE3 and later increased expression of this transporter. In contrast, NHE2 activity is not regulated by AII. NHE2 has a lesser contribution to intestinal Na^+ ^transport [32] and is regulated differently by agonist-activated second messenger pathways [24].

The actions of AII on intestinal epithelial Na transport appear to involve both acute and chronic effects. Acutely, AII rapidly stimulates insertion of NHE3 into the apical membrane which increases Na^+ ^absorption. However, over time, AII stimulates transcriptional activation of the NHE3 gene, resulting in increases in total NHE3 protein abundance. This second phase is associated with additional apical membrane insertion of NHE3 and enhanced Na absorptive capacity. The effects of AII on intestinal transport could be either indirect and/or direct and the present studies demonstrate that direct effects occur. When AII was given intravascularly, it stimulated the enteric nervous system which could affect intestinal electrolyte transport [11,12]. However, when AII was added to *in vitro *preparations of rat small intestine serosally, it stimulated Na^+ ^transport, suggesting that AII was acting directly on epithelial cells [13] similar to present results in mouse jejunum. Adding further support to this possibility, AII binds with high affinity to intestinal enterocyte membranes [18,33]. More recent studies of porcine jejunum found both types of the AII receptor, however full thickness intestine was used for analysis which contains both epithelial and non-epithelial cell components [15]. Luminal/brush border type I and II AII receptors have been functionally demonstrated in intestine where luminal AII, through the type I receptor, inhibits apical Na^+^-dependent glucose transport [33]. A similar finding of brush border type I AII receptor regulates apical glucose uptake in LLC-PK porcine kidney cells [34]. The effects on apical sodium transport were not measured in these studies of intestinal and renal brush border membranes.

The intestine possesses a complete renin-angiotensin system [35] that appears to have local autocrine and paracrine effects [36]. Angiotensinogen, renin and the angiotensin converting enzyme are all expressed in the intestine, as well as both types of AII receptors [35,37-39]. From the present studies, we cannot determine the role of systemic versus locally produced AII. Indeed, regulation of these 'local' renin-angiotensin systems has received modest investigation. In the intestine, activity of the Na^+^-dependent glucose transporter, but not leucine transporter, was decreased by AII, an effect that may be related to its effects on the brush border [33].

The present studies strongly support the type I receptor as the mediator of the AII effects on the acute stimulation of NHE3. The signal transduction pathways of the type I AII receptor are complex and involve multiple pathways. In a cultured small intestinal cell line, IEC-6, AII stimulates several transduction pathways including phospholipase D, certain isoforms of protein kinase C, and activation of the EGF receptor [19-21] that stimulate cell growth.

## Conclusion

AII can directly stimulate intestinal epithelial Na^+ ^absorption through the AII-receptor activation of several key signaling pathways that induce acute and chronic changes in NHE3 membrane trafficking and gene transcription. These processes involve rapid exocytosis of the major non-nutrient Na^+ ^absorptive pathway, NHE3 via activation of the type I receptor and activation of complex transduction pathways. AII does not, however, stimulate exocytosis and activity of the related exchanger NHE2. AII activation of the intestinal epithelial cells also has more prolonged effects on fluid and electrolyte absorption and homeostasis as expression of the exchanger NHE3 is increased. We conclude that angiotensin II has a direct role in regulating intestinal fluid and electrolyte absorption which may contribute to its overall effects in regulation systemic volume and blood pressure.

## Methods

### Cell Culture

Caco-2BBE intestinal epithelial cells, provided by Dr. Mark Mooseker [40](Yale University, New Haven, CT), were grown as confluent monolayers on rat tail collagen-coated Transwells in DMEM supplemented with 10% vol/vol fetal bovine serum, 2 mM glutamine, 10 μg/ml transferrin, 50 U/ml penicillin, and 50 μg/ml streptomycin in a humidified atmosphere of air containing 5% CO_2_. Cells were seeded onto the collagen coated Transwells at a density of 10^5 ^cells/cm^2 ^and cultured for 14 days before each experiment. Differentiation of Caco-2BBE cells in culture was determined by expression of villin and alkaline phosphatase.

### Apical membrane unidirectional ^22^Na^+ ^influx as a measure of Na^+^-H^+ ^exchange (NHE) activity

For influx studies, Caco2BBE cell monolayers were washed once in 150 mM choline Cl, 10 mM HEPES pH 7.4 and then unidirectional apical membrane sodium uptakes were determined in flux buffer (130 mM Choline Cl, 5 mM KCl, 1 mM MgCl_2_, 2 mM CaCl_2_, 15 mM HEPES pH 7.4, 20 mM NaCl with 1 μCi/ml [^22^NaCl]) for ten minutes. Sodium influx was stopped by 4 washes in cold buffer (140 mM NaCl, 5 mM KCl, 15 mM HEPES pH 7.4 and 1 mM Na_3_PO_4_) and was calculated by dividing the accumulated DPM by the specific Na activity in the medium. Dimethylamiloride (DMA) (500 μM) and HOE 694 (30 μM) were used to distinguish NHE2 and NHE3 activities, as previously described [24]. NHE2 and NHE3 activities were defined as the HOE694-sensitive and -insensitive components of total DMA-inhibited unidirectional ^22^Na influx, respectively.

For studies on apical NHE3 exocytosis, cell monolayers were stimulated with AII for varying times with or without pretreatment with inhibitors as designated. AII was added directly into the basolateral medium. Monolayers were rapidly cooled by placing on ice, changing medium to phosphate buffered saline with 0.5 mg/ml sulfo-NHS biotin only on the apical side. Monolayers were incubated for 30 min with the apical biotinylation solution. Over this period, we had previously shown that biotinylation of basolateral and intracellular proteins does not occur [25]. Biontinylation was terminated by the addition of 10 μl of 1 M Tris pH 8.0 which will reacts rapidly with all free biotin. Cell monolayers were scraped off the filters, pelleted and resuspended in immunoprecipitation buffer (IP buffer, composition in mmol/l:150 NaCl, 10 HEPES pH 7.4, 2 EDTA, 1 PMSF, 0.1% SDS 0.5% deoxycholate (both wt/vol) and 1% Triton X-100 (vol/vol)). Samples were solubilized, an aliquot removed to measure protein and total NHE3, and, to the remainder. streptavidin-agarose was added. Samples were rotated for 120 min, washed 3 times with IP buffer, and samples eluted by boiling in 1× Laemmli buffer. Biotinylated apical surface proteins as well as total NHE3 were analyzed by Western blotting.

Total cellular protein (35 μg) or IP samples were separated on 7.5% SDS-PAGE and immediately transferred to PVDF membranes (Polyscreen, Perkin Elmer Life Sciences, Boston, MA) in 1× Towbin's buffer (25 mM Tris, 192 mM glycine, pH 8.8 with 15% vol/vol methanol). Membranes were blocked in T-TBS (Tween 20-Tris buffered saline; 150 mM NaCl, 5 mM KCl, 10 mM Tris pH 7.2 with 0.1% vol/vol Tween 20) containing 5% wt/vol nonfat dry milk (Carnation, Solon, OH) for 60 min at room temp. Blots were incubated overnight at 4°C with affinity purified specific rabbit polyclonal antisera to NHE2 and NHE3 developed and characterized by our laboratory [41,42]. Blots were developed using an enhanced chemiluminescence system (Supersignal, Pierce Chemical, Rockford, IL).

### RNA isolation, reverse transcription, and real-time PCR

Caco-2BBE cells were treated with AII (1 nM) for varying times. Total RNA was isolated using TRIzol reagent (Invitrogen). One μg RNA was reverse transcribed by random priming (Superscript II RT, Invitrogen, Carlsbad, CA) and one twentieth used for real-time PCR performed on an I Cycler (BioRad, Hercules, CA) using SybrGreen Mix and primers for human NHE3 (NM_004171, bases 2062–2164 and GAPDH (NM_002046, bases 160–229). Relative mRNA levels were calculated using the comparative threshold cycle (ΔΔC_t_) method [43]. Each PCR reaction was performed in triplicate, and all experiments were repeated three times. For each sample, mRNA levels of both NHE3 and GAPDH were measured and the cycle threshold of NHE3 subtracted from that of GAPDH. This value was set to one for untreated control conditions at zero time and other time points are calculated relative to this change. For analysis of AII receptor type, one twentieth of the reverse transcription reaction was used for amplification with primers for human ATGR1 (NM_004835, bases 675–808) and AGTR2 (NM_000686, bases 740–918). PCR reactions were amplified for 30 cycles and the PCR products were first analyzed by agarose gel for confirmation of correct size and then subcloned into pCR2.1-TOPO (Invitrogen) and sequenced.

### Luciferase reporter activity

A 2200 bp region of the rat NHE3 promoter [26] was a generous gift of Dr. A. Cano (Univ. of Texas Southwestern Medical Center, Dallas, TX). This promoter was linked to firefly luciferase in the plasmid pGL3 (Promega, Madison, WI). Monolayers were transiently transfected with 2 μg of the NHE3 promoter-firefly luciferase plasmid along with 100 ng of a thymidine kinase promoter linked to Renilla luciferase reporter plasmid using 10 μl of the transfection reagent LT-1 (Mirus, Madison, WI) according to the manufacturer's directions. Twenty four hours after transfection, monolayers were treated with AII. Cell monolayers were harvested in lysis buffer provided with the dual-luciferase assay kit and firefly and Renilla luciferase measured in a Berthold Lumat luminometer (Berthold, NJ) using the protocol provided with the Dual Luciferase assay system (Promega, Madison, WI).

### Statistical analysis and densitometry

For all statistical comparisons, Instat software for the Macintosh (GraphPad, San Diego, CA) was used. For multiple comparisons, analysis of variance using a Bonferroni correction for the number of comparisons was used. For paired comparisons, a paired Student's T test was used. To quantitate differences in images of protein or mRNA expression, films were scanned and densitometry performed using NIH Image 1.54 software.

## Authors' contributions

MWM designed, performed the experiments, analyzed data, and wrote the manuscript. YL and EBC conceived and designed the experiments and revised the manuscript. All authors read and approved the final manuscript.
